# ISLAND Campus: a fee-free formal university educational intervention in mid- to later-life to reduce modifiable risk factors for dementia and improve cognition

**DOI:** 10.3389/fnagi.2024.1479926

**Published:** 2024-12-05

**Authors:** Eddy Roccati, Alex Kitsos, Aidan David Bindoff, Jane Elizabeth Alty, Larissa Bartlett, Jessica Marie Collins, Anna Elizabeth King, Hannah Fair, Kathleen Doherty, James Clement Vickers

**Affiliations:** ^1^Wicking Dementia Research and Education Centre, University of Tasmania, Hobart, TAS, Australia; ^2^Royal Hobart Hospital, Hobart, TAS, Australia

**Keywords:** education, modifiable risk factors, cognition, biomarkers, intervention, longitudinal, epidemiology, public health

## Abstract

**Introduction:**

Previous research has tended to focus on early-life education for dementia risk reduction, yet there are great gains for building cognitive reserve in mid- to later-life through educational interventions. ISLAND (Island Study Linking Ageing and Neurodegenerative Disease) Campus offered free university study to all ISLAND participants, with flexible in-person/online learning models to remove educational, socioeconomic and geographical barriers. Here the core hypothesis of ISLAND Campus was investigated: that engagement in later life education leads to improvements in modifiable risk factors for dementia, cognition and blood-based biomarkers.

**Methods:**

ISLAND Campus participants were matched on age and gender to non-Campus participants via propensity score method, with optimal matching based on logistic regression. Participants completed online surveys on health, demographics, modifiable dementia risk factors via a customized Dementia Risk Profile (DRP) tool and provided blood samples for APOE genotyping and plasma phosphorylated-tau (p-tau). Cognition was measured online via the validated Cambridge Neuropsychological Test Automated Battery Paired Associates Learning (PAL) and Spatial Working Memory (SWM) tasks. Impact of the opt-in formal educational intervention was tested in R via ANCOVA.

**Results:**

Total participants were 986 (interventio*n* = 492, control = 492), mean age of 61.2 years, 73.2% female, 11.7 mean years of education and 25.0% APOE e4+. Over 4 years of follow-up, intervention participants significantly improved working memory (SWM) and their risk factor profiles as measured via the DRP (*p* < 0.001), indicating a significant change towards lower dementia risk. Intervention and control participants were similar on socioeconomic status, location of residence, p-tau and APOE e4 presence, however Campus participants displayed a significantly higher proportion of prior university study completion (76.0%) than controls (60.0%). Intervention participants enrolled in a variety of university degrees, the most common were Diploma of Family History (*n* = 103, 20.9%), Diploma of Arts (*n* = 74, 15.0%) and Diploma of Fine Arts (*n* = 52, 10.5%).

**Discussion:**

ISLAND Campus has shown how free later-life university education was associated with improvements in modifiable dementia risk factors over time and cognition. Given opt-in intervention participants were significantly more likely to have a prior university education, later life formal educational interventions should be targeted at individuals with lower prior education.

## Background

Fewer years of education in early-life is a substantial contributor to dementia risk (Livingston et al., 2020, 2024), yet there is limited evidence for targeting mid- to later-life education in primary prevention policies (Collins et al., 2019). Single-domain interventions targeting cognitively stimulating interventions have demonstrated inconsistent results (Wolinsky et al., 2013; Ball et al., 2002), likely due to a shorter duration of intervention (weeks to months), explaining initial post-intervention benefits that were unsustainable at follow-up (Wolinsky et al., 2013; Ball et al., 2002; Kivipelto et al., 2020). Multidomain trials are typically longer in duration (up to 2 years) (Ngandu et al., 2015), however, they tend to use older cohorts (Bäckman et al., 2004) and potentially confound informal cognitive training with concomitant interventions targeting hypertension, cardiovascular health, physical activity and diet (Ngandu et al., 2015). Further, research to date has focussed mainly on formal education in childhood and early adulthood, neglecting the potential for older adults to gain neuroprotection and build cognitive reserve at a critical period in their lives, as well as the added benefit from formal education as a contributor to motivation to change lifestyle behaviors toward better brain health (Siette et al., 2023).

Educational attainment is recognized as a fundamental determinant of health (Phelan et al., 2010), and we now know that formal education is a promising autonomous and sustainable strategy to improve health in mid- to later-life (Yamashita et al., 2019). Despite the preponderance of studies investigating education in early-life, there is growing evidence that education later in life can reduce dementia risk, even for people with low educational attainment in their earlier years (Najar et al., 2019). This benefit is hypothesized to be attributable to an increase in cognitive activity that subsequently boosts an individual’s cognitive reserve, or their resistance to neuropathological insult throughout the course of ageing (Alty et al., 2023; Hill et al., 2021). Education is multifaceted, and previous research has demonstrated how lower education is linked to reduced brain volume (Gonzalez-Gomez et al., 2024), reduced connectivity (Montemurro et al., 2023) and ultimately an increased risk for dementia (Livingston et al., 2024). Given this, existing socioeconomic inequities and life course inequality are likely contributing to disparities in educational attainment (Hilal and Brayne, 2022; Walhovd et al., 2022), and subsequent brain health outcomes (Hatzenbuehler et al., 2024).

In the Australian island state of Tasmania, research has demonstrated the feasibility and efficacy of mid- to later-life formal educational interventions, both to improve cognition (Bindoff et al., 2021) and improve modifiable risk factor adherence (Bartlett et al., 2023). The Tasmanian Healthy Brain Project (THBP) was a prospective, longitudinal investigation into the impact of mid- to later-life tertiary education on cognitive decline in adults aged 50–79 years of age at baseline in 2010–2011 (Summers et al., 2013). Participants were offered subsidized educational enrolment fees, and were invited for biennial neuropsychological evaluation (Summers et al., 2013). Over 10 years, participation in the THBP’s formal tertiary educational intervention was significantly associated with improved cognitive trajectories, particularly in domains of language and verbal/episodic memory (Bindoff et al., 2021).

To build on these results, we designed a prospective longitudinal follow-up study: ‘ISLAND Campus’. ISLAND (Island Study Linking Ageing and Neurodegenerative Disease) is a longitudinal study of over 14,000 participants (Bartlett et al., 2021) that has a public health focus with the ultimate goal of reducing dementia incidence in Tasmania. Based around the model of the THBP, ISLAND Campus provided the opportunity for participants to engage in further study in a select group of courses at no cost. ISLAND Campus expanded on THBP’s foundation by removing several barriers to participation, such as being completely fee-free for participants, open to any Tasmanian over 50 years of age and offering flexible in-person/online learning models that endeavored to remove geographical and socioeconomic barriers to participation. ISLAND Campus participants also completed a battery of surveys relating to dementia risk, such as the Dementia Risk Profile (DRP), a traffic-light personalized report on individual modifiable risk factors based on WHO (2019) dementia guidelines.

Our ultimate aims with ISLAND Campus are to determine the impact of mid- to later-life university education on modifiable dementia risk behaviors, cognitive function and plasma biomarkers. ISLAND Campus will delve deeper than the THBP, by understanding the unique intentions, expectations and barriers to participation in later life education, as well as incorporating an array of covariates that may mediate this relationship, such as perceived stress, general self-efficacy and health literacy. Here we present pilot results from the ISLAND Campus sub-study. We hypothesized that those who have taken up the university level education as part of ISLAND Campus will demonstrate improvements in key measures of dementia risk compared with their non-intervention counterparts in the general ISLAND cohort; higher cognitive scores, lower plasma concentrations of p-tau 181 and lower DRP profile scores.

## Methods

### Study population

This longitudinal observational study included 984 participants recruited from ISLAND which began recruitment in October 2019 and was open to all people living in Tasmania over the age of 50 years (Bartlett et al., 2022). No other inclusion or exclusion criteria was set. ISLAND Campus was initially offered to all ISLAND participants in the second semester of 2020. Between June and September 2020, full fee-waiver scholarships were made available by University of Tasmania (UTAS) to study participants to undertake a full university course of their choosing. ISLAND Campus participants were recruited via email and the ISLAND Home Portal, where their status as an ISLAND participant was confirmed with UTAS’ Division of Future Students to apply the fee-free waiver. Campus was available to all ISLAND participants, regardless of geographical location, socioeconomic status or previous educational attainment. To be eligible for Campus, ISLAND participants needed to provide additional mandatory survey data in addition to the general ISLAND protocol (Bartlett et al., 2022). As of October 1, 2022, a total of 13,822 had registered interest in the main ISLAND study, of whom 1,487 participants consented to Campus and 783 were deemed eligible to receive a fee-free UTAS course. Of the 783 ISLAND participants eligible to receive a full fee waiver for their UTAS course, 596 enrolled in a course/unit from Semester 2, 2020 onwards and 492 completed a course/unit as part of the ISLAND Campus initiative. These 492 participants who completed a course/unit as part of Campus were matched with 492 who did not consent to Campus. Group differences between all ISLAND participants with available data (*n* = 3,249) and ISLAND Campus (*n* = 492) are displayed in Supplementary Table 1. Participant flow diagram in CONSORT (Moher et al., 2010) format is displayed in Supplementary Figure 1.

### Survey materials

ISLAND surveys used in this analysis were Background Health Survey and DRP (Bartlett et al., 2022). Using pre-defined cut-offs for body mass index (BMI), physical activity, cognitive activity, alcohol consumption, diet, smoking and cardiometabolic health; the DRP calculates an individual’s risk as low (green), medium (orange) or high (red) for each domain (Roccati et al., 2023). ISLAND Campus student enrolment and completion data was provided by UTAS’ Division of Future Students. In addition to the general ISLAND surveys, participants completed several further validated psychometric surveys such as the Perceived Stress Scale (PSS) (Cohen et al., 1983), New General Self-Efficacy Scale (NGSS) (Chen et al., 2001), All Aspects of Health Literacy Scale (AAHL) (Chinn and McCarthy, 2013).

### Cognition

Cognitive function was longitudinally measured in 2021 and 2023 using validated tests from the Cambridge Neuropsychological Test Automated Battery (Robbins et al., 2004) (CANTAB). Two cognitive domains were assessed remotely via the online ISLAND Home Portal: Paired Associates Learning (PAL) assessed episodic memory through the learning and recall of visual information over successive trials and has been shown to be sensitive to cognitive decline in early AD (Swainson et al., 2001); Spatial Working Memory (SWM) assessed executive function, via the retention and manipulation of visuospatial information. PAL Total Errors (Adjusted) indicates the number of times a participant chose the incorrect box for a stimulus on assessment problems (PALTE), plus an adjustment for the estimated number of errors they would have made on any problems, attempts and recalls they did not reach. This measure allows performance comparisons on errors made across all participants regardless of those who terminated early versus those completing the final stage of the task. SWM Strategy indicates the number of times a participants begun a new search pattern from the same box they started with previously. If they always begun a search from the same starting point, we inferred that the participant is employing a planned strategy for finding the tokens. Therefore, a low score indicates high strategy use (1 = they always begin the search from the same box), and a high score indicates that they are beginning their searches from many different boxes. This score is calculated across assessed trials with 6 tokens or more.

### Plasma biomarkers

All ISLAND participants were invited to provide a blood sample at in-person clinics geographically dispersed across Tasmania held throughout 2021. Participants booked in for collection via the ISLAND Home Portal and were asked to avoid exercise in the 24 h prior to their collection to prevent acute increases in circulating proteins. After providing in-person consent, blood samples were collected by trained phlebotomists via venepuncture into 2 × 8.5 mL tubes: 1 × gold serum separator tube vacutainer for serum; 1 × purple ethylenediaminetetraacetic acid vacutainer for plasma. The plasma tubes were centrifuged at 2000’g for 10 min at 4°C, and the resulting plasma aliquoted into screw-top polypropylene tubes and stored in −80°C freezers within 2 h of collection. Prior to cross-sectional p-tau 181 analysis, plasma sample aliquots were removed from −80°C storage and kept at room temperature for 1 h to thaw. Samples were then mixed by vortexing for approximately 10 s and then centrifuged at 10,000’g for 5 min at room temperature to remove particulate matter. Plasma p- tau 181 was measured using the commercially available Quanterix pTau181 Advantage V2 Single Molecule Array (Simoa) Kit (Cat no. 103714) on the Quanterix SR-X biomarker system. Briefly, the kits were removed from 4°C and allowed to warm to room temperature for 30–60 min. The resorufin-*β*-D-galactopyranoside (RGP) reagent was prepared by placing it on the Quanterix Plate Shaker at 800 rpm for 25 min at 35°C. The assay was performed as per manufacturer’s instructions, with the supplied calibrators and high and low control samples run in duplicate and participant plasma samples run in single. Fitted concentrations are provided, as interpolated from the calibration curve fitted by the Quanterix SR-X biomarker system software. The intraassay coefficient of variation CV for the high and low control samples were 2.65 and 4.18%, respectively.

### Statistical analysis

Statistical analyses were completed in R (version 4.3.1) (R Core Team, 2023), where 2-sided *p* values <0.05 were considered statistically significant. Due to the potential imbalances between intervention (ISLAND Campus) and control (ISLAND non-Campus) participants, we conducted propensity score matching using the ‘matchit’ package for R (Stuart et al., 2011), a commonly used method to account for non-random group assignment in observational studies (Olmos and Govindasamy, 2015). Propensity scores are the conditional probability of assignment to a treatment condition given a set of observed covariates: e = p (z = i|χ^2^) (Olmos and Govindasamy, 2015). They provide a powerful method of matching an intervention group (*n* = 492 Campus) with a control group drawn from a pool of potential control group subjects (*n* = 3,249 ISLAND). ISLAND Campus participants (*n* = 492) were matched on age and gender to non-Campus participants (*n* = 492) via propensity score matching method, with optimal matching based on logistic regression. A propensity score model using age and gender was selected due to showing the best model fit (lowest Akaike Information Criterion) between control and intervention covariate distribution. Group balance hypothesis testing was conducted using *χ* (Collins et al., 2019) test for categorical variables and 1-way analysis of variance (ANOVA) test for continuous variables. Multiple generalized additive regression models were conducted to analyze the impact of the intervention (post-exposure ~ intervention + pre-exposure + covariates + error) on modifiable risk factors for dementia as measured by the DRP, cognition measured via CANTAB and plasma concentrations of p-tau 181. For a robust assessment of the impact of the ISLAND Campus intervention (post-exposure), models adjusted for propensity score weight (PSW) as well as baseline data (pre-exposure) and covariates for longitudinal analysis. Model assumptions were tested via ‘performance’ (Lüdecke et al., 2021), summary results prepared using ‘stargazer’ (Hlavac, 2018) packages for R. Trajectories were projected using generalized additive models with thin plate regression splines. Model 1 was unadjusted. Model 2 adjusted for age, gender and education. Model 3 adjusted for age, gender, education, baseline values and PSW. Multiple comparisons were corrected for using the Bonferroni method. To ensure our study was sufficiently powered to detect the observed effect size of the ISLAND Campus intervention, a *post-hoc* power analysis was conducted using the ‘pwr’ package for R (Champely et al., 2017).

### Ethics statement

All ISLAND Campus participants completed informed consent prior any data collection activity. ISLAND Campus is a core sub-study of ISLAND and has been approved by UTAS’ Health and Medical Human Research Ethics Committee (HREC H001864). All procedures were carried out in accordance with the National Health and Medical Research Council’s National Statement on Ethical Conduct in Human Research and the Declaration of Helsinki.

## Results

Out of 3,249 ISLAND participants, a total of 984 (interventio*n* = 492, control = 492) matched via propensity score method were included in the current study (mean age of 61.1 years, 73.2% female, 11.6 mean years of education, 23.9% APOE e4+). Intervention and control participants were similar on socioeconomic status, location of residence and APOE e4 presence; however intervention participants had significantly higher prevalence of prior university study completion (76.0%) than controls (60.0%). Demographics of these participants compared with the non-Campus ISLAND participants are presented in Table 1. Intervention participants enrolled in a variety of university degrees, the most common were Diploma of Family History (*n* = 103, 20.8%), Diploma of Arts (*n* = 74, 15.0%) and Diploma of Fine Arts (*n* = 52, 10.6%). These diplomas are equivalent to 1 year of full-time study. A full course enrolment list is provided in Table 2.

**Table 1:** Demographics of the cohort.

	Campus participant (*N* = 492)	Not campus participant (*N* = 492)	*P*-value
**Age (in years)**			0.859
Mean (SD)	61.2 (7.31)	61.1 (7.41)	
Median [Min, Max]	60.0 [50.0, 86.0]	60.0 [50.0, 89.0]	
**Gender**			0.329
Female	351 (71.3%)	369 (75.0%)	
Male	138 (28.0%)	122 (24.8%)	
Other	2 (0.4%)	0 (0%)	
Prefer not to say	1 (0.2%)	1 (0.2%)	
**IRSAD decile**			0.057
Mean (SD)	5.43 (2.84)	5.08 (2.90)	
Median [Min, Max]	6.00 [1.00, 10.0]	6.00 [1.00, 10.0]	
**Remoteness area**			0.467
Inner Regional Australia	368 (74.8%)	350 (71.1%)	
Outer Regional Australia	118 (24.0%)	138 (28.0%)	
Remote Australia	3 (0.6%)	2 (0.4%)	
Very Remote Australia	2 (0.4%)	1 (0.2%)	
**Total years of school**			0.177
Mean (SD)	11.7 (1.34)	11.6 (1.58)	
Median [Min, Max]	12.0 [4.00, 20.0]	12.0 [3.00, 20.0]	
**Highest level of education obtained**			**<0.001**
Bachelor’s degree	121 (24.6%)	110 (22.4%)	
Certificate or apprenticeship (including Cert 2, 3 or 4)	37 (7.5%)	60 (12.2%)	
Diploma / Associate degree	73 (14.8%)	91 (18.5%)	
High School	26 (5.3%)	62 (12.6%)	
Higher University degree (Honours, Graduate Diploma, Masters or PhD)	215 (43.7%)	156 (31.7%)	
Other	16 (3.3%)	9 (1.8%)	
**Prior completion of tertiary education**			**<0.001**
No	117 (23.8%)	192 (39.0%)	
Yes	374 (76.0%)	295 (60.0%)	
**Have you noticed a substantial change in your memory and mental function in recent years?**			0.339
No	400 (81.3%)	388 (78.9%)	
Yes	88 (17.9%)	101 (20.5%)	
**Is there a history of conditions such as dementia in your direct family for example siblings, parents, grandparents, aunties and uncles?**			0.447
No	272 (55.3%)	257 (52.2%)	
Yes	217 (44.1%)	228 (46.3%)	
**Apolipoprotein E (APOE) e4 presence**			0.669
No	180 (36.6%)	105 (21.3%)	
Yes	57 (11.6%)	38 (7.7%)	

Displayed are group differences, means and SD or *N* (%) with participants grouped on propensity score method in ISLAND campus. *p*-values indicate chi-squared for categorical, and ANOVA for continuous measures.

Bold values indicate statistical significance (*p* < 0.005). IRSAD, index of relative socio-economic advantage and disadvantage, SD, standard deviation, APOE, apolipoprotein E; PhD, doctor of philosophy.

**Table 2:** ISLAND Campus course enrolment.

**Course name**	**N**	**%**
Diploma of Family History	103	20.9
Diploma of Arts	74	15.0
Diploma of Fine Arts	52	10.5
Diploma of General Studies	38	7.7
Diploma of Sustainable Living	37	7.5
Diploma of Languages	29	5.9
Bachelor of Psychological Science	19	3.9
Diploma of Creative Arts and Health	18	3.7
Bachelor of Dementia Care	17	3.4
Bachelor of Science	11	2.2
Bachelor of Business	9	1.8
Bachelor of Design	8	1.6
Bachelor of Natural Environment and Wilderness Studies	7	1.4
Diploma of Dementia Care	7	1.4
Diploma of Music	7	1.4
Bachelor of Information and Communication Technology	6	1.2
Undergraduate Certificate in Sustainable Living	5	1.0
Unistart Program	5	1.0
Associate Degree in Applied Business	4	0.8
Associate Degree in Applied Health and Community Support	4	0.8
Bachelor of Architecture and Built Environments	4	0.8
Associate Degree in Applied Science	3	0.6
Associate Degree in Applied Technologies	3	0.6
Diploma of University Studies	3	0.6
Bachelor of Global Logistics and Maritime Management	2	0.4
Bachelor of Natural Environment and Wilderness	2	0.4
Associate Degree in Agribusiness	1	0.2
Bachelor of Agricultural Science	1	0.2
Bachelor of Agricultural Science with Honours	1	0.2
Bachelor of Applied Science (Professional Honours in Environmental Management)	1	0.2
Bachelor of Arts	1	0.2
Bachelor of Engineering (Specialisation) with Honours	1	0.2
Bachelor of Fine Arts	1	0.2
Bachelor of Health and Human Services (Leadership) Professional Honours	1	0.2
Bachelor of Marine and Antarctic Science	1	0.2
Bachelor of Medicines Management with Professional Honours in Complementary Medicines	1	0.2
Diploma of Fine Arts and Design	1	0.2
Diploma of Pharmacy Studies	1	0.2
Graduate Certificate in Business Studies	1	0.2
Graduate Diploma in Counselling	1	0.2
Master of Business Administration	1	0.2
Master of Information Technology and Systems	1	0.2

Displayed are the individual course names offered as part of ISLAND Campus and the number of participants (with %) enrolled in each. Data presented was extracted from UTAS’ division of future students.

### Modifiable dementia risk behaviors

**Figure 1:**
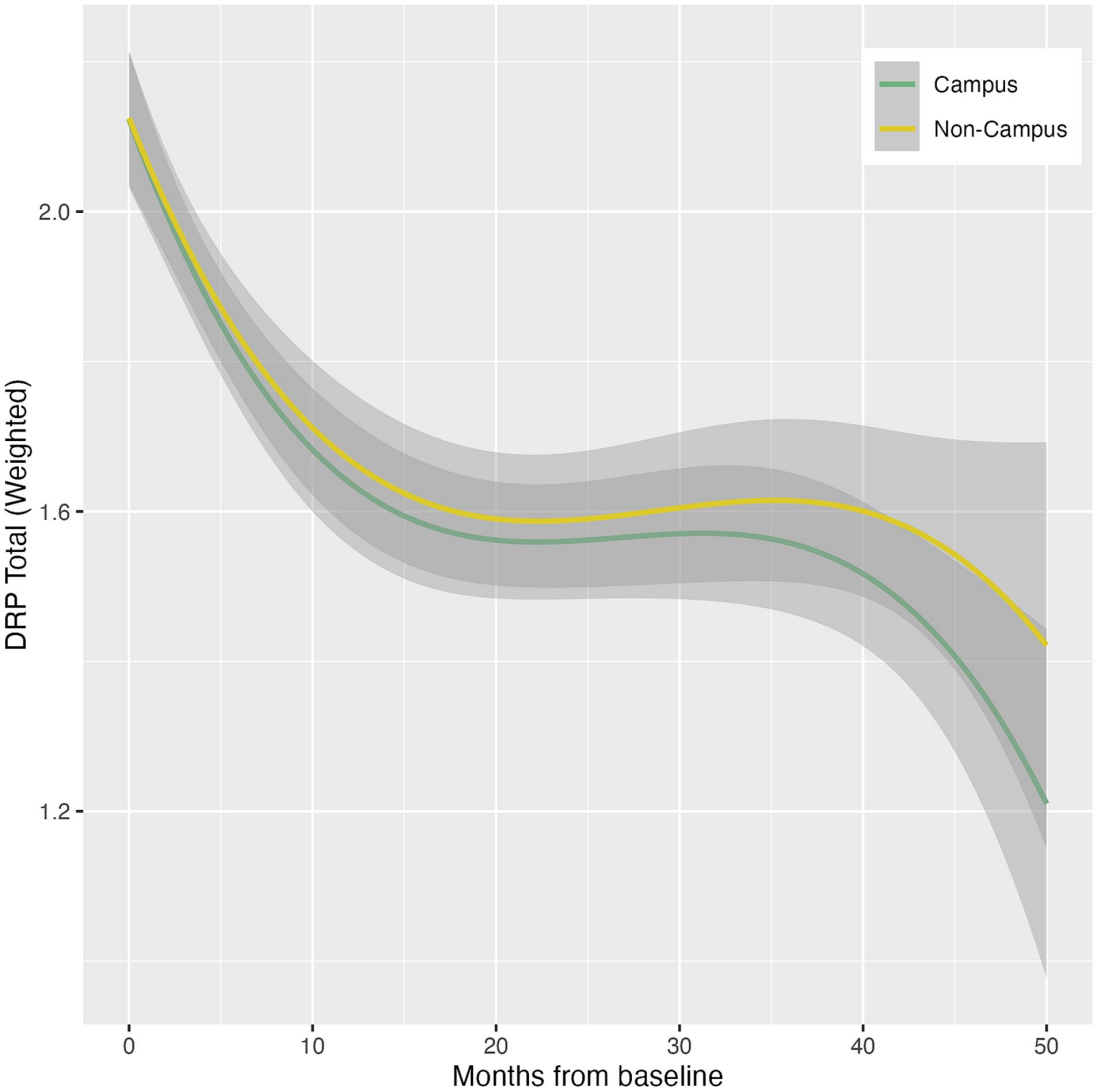
Total change in DRP between intervention (Campus) and control (Non-campus) participants in the ISLAND Campus study. For each DRP category (low, medium and high) delta scores indicate longitudinal change (Follow up – Baseline) with a negative result indicating movement toward a lower DRP total score.

**Figure 2:**
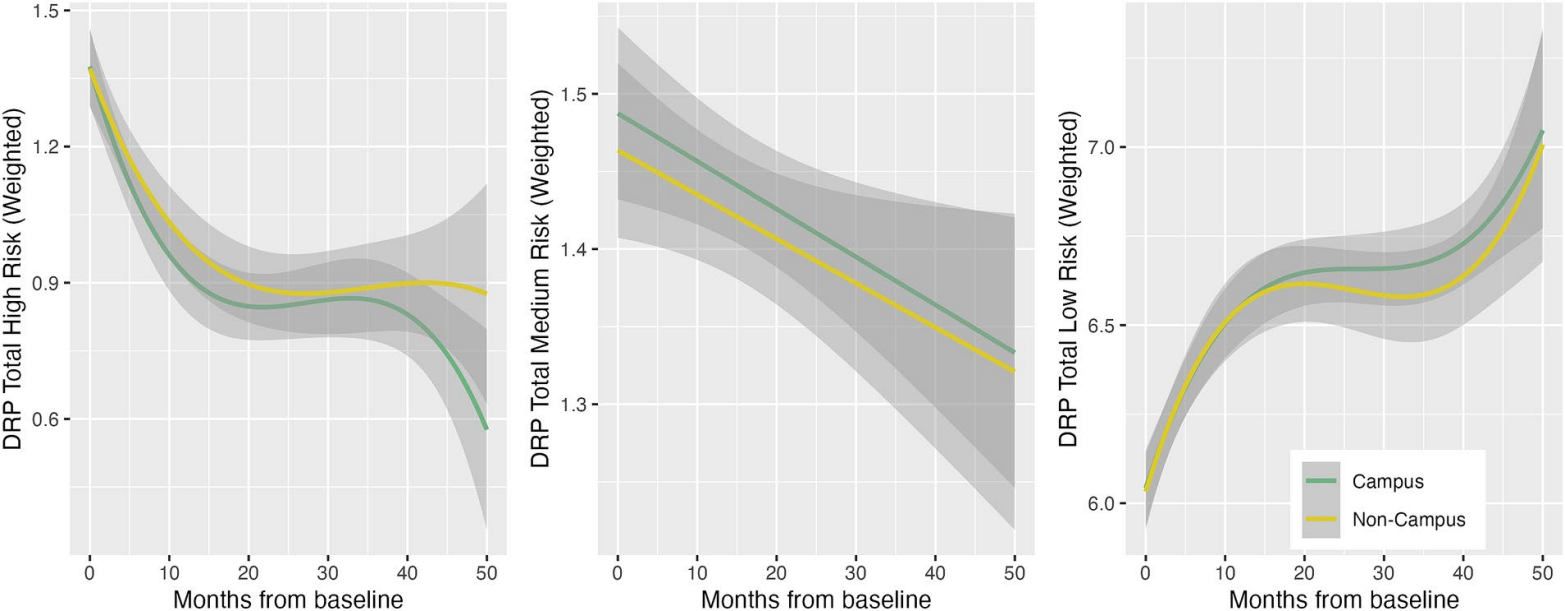
Longitudinal total DRP score trajectories between intervention (Campus) and control (Non-campus) participants in the ISLAND Campus study. Trajectories are generalized additive models with penalty-based smoothing regression splines.

**Figure 3:**
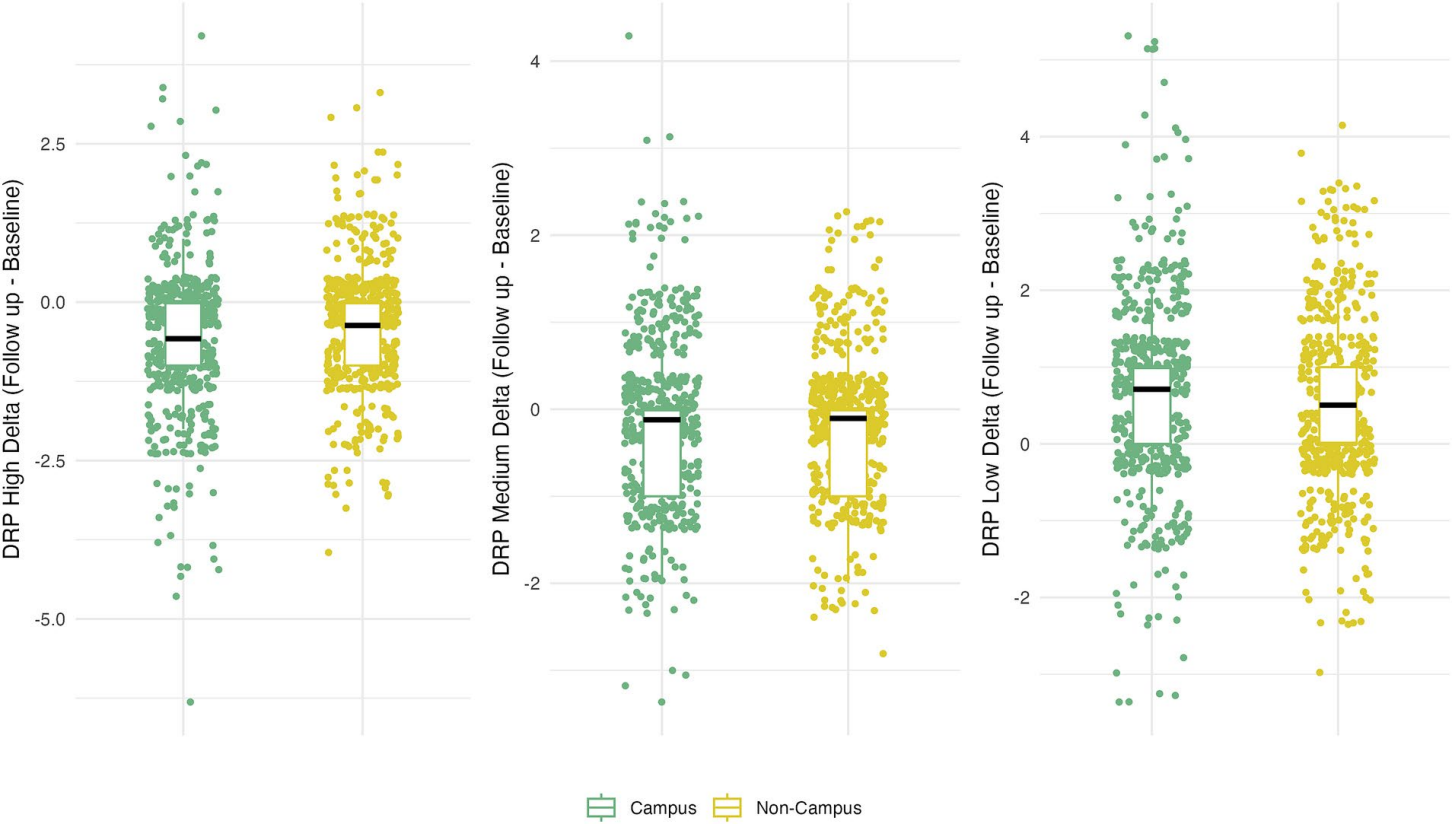
Longitudinal low, medium and high DRP score trajectories between intervention (Campus) and control (Non-campus) participants in the ISLAND Campus study. Trajectories are generalized additive models with thin plate regression splines, accounting for PSW.

Participation in ISLAND Campus had a significantly positive impact on total DRP change, with those in the intervention group displaying greater improvements on total DRP over time (Table 3; Figure 1). This observation remained after controlling for covariates of age, gender, prior tertiary education, baseline DRP and PSW. At baseline, participants were comparable in terms of modifiable risk factors as measured by the DRP (Supplementary Table 2). At follow-up, ISLAND Campus participants displayed significantly lower BMI risk and lower cognitive activity risk compared with their non-Campus counterparts (Supplementary Table 2). Graphed longitudinally, ISLAND Campus participants showed movement toward lower total scores on total DRP (Figure 2), as well as when split into low, medium and high score categories on the DRP (Figure 3).

**Table 3:** Impact of ISLAND campus intervention on DRP.

	Dependent variable:		
	**Total DRP at follow up**		
	(1)	(2)	(3)
Campus participation (Non-campus)	0.212***	0.186**	0.215***
	*p* = 0.005	*p* = 0.012	*p* = 0.0004
Age		−0.038***	0.136
		p = 0.000	*p* = 0.381
Gender (male)		0.276***	−0.857
		*p* = 0.001	*p* = 0.411
Prior completion of tertiary education		−0.166**	−0.012
		*p* = 0.037	*p* = 0.857
Total DRP at baseline			0.543***
			p = 0.000
PSW			−20.388
			*p* = 0.330
Constant	1.552***	3.901***	3.869
	p = 0.000	*p* = 0.000	*p* = 0.152

Observations	984	978	978
*R* ^2^	0.008	0.073	0.388
Adjusted *R*^2^	0.007	0.067	0.383
Residual Std. Error	1.168 (df = 982)	1.133 (df = 971)	0.922 (df = 969)
F Statistic	8.138^***^ (df = 1; 982) (*p* = 0.005)	12.668^***^ (df = 6; 971) (*p* = 0.000)	76.766^***^ (df = 8; 969) (*p* = 0.000)

Testing the fee-free educational intervention on longitudinal changes in total scores on the dementia risk profile (DRP).

*p < 0.1; **p < 0.05; ***p < 0.01. Unstandardized coefficient estimates are presented, with raw *p*-values provided below. For categorical variables; non-campus, prior completion of tertiary education and males are effect size estimates. PSW, propensity score weighting.

### Cognitive function

ISLAND Campus participation was not significantly associated with longitudinal change in CANTAB PAL (Table 4), and this association remained non-significant after adjusting for covariates. Between baseline and follow up, ISLAND Campus participants significantly improved their spatial working memory (SWM). This effect remained after controlling for covariates such as age, gender, prior tertiary education, baseline CANTAB SWM and PWS (Table 5).

**Table 4:** Impact of ISLAND campus intervention on cognition via CANTAB paired associates learning (PAL).

	Dependent variable		
	**PAL total errors adjusted (2–8) at follow up**		
	(1)	(2)	(3)
Campus participation (Non-campus)	1.148	1.105	0.827
	p = 0.355	*p* = 0.359	*p* = 0.438
Age		0.392***	−2.365
		*p* = 0.00001	*p* = 0.398
Gender (male)		3.025**	19.58
		*p* = 0.022	*p* = 0.297
Prior completion of tertiary education		−1.712	−1.411
		*p* = 0.174	*p* = 0.206
PAL total errors adjusted (2–8) at baseline			0.393***
			*p* = 0.000
PSW			356.752
			*p* = 0.344
Constant	12.427***	−11.688**	−55.475
	*p* = 0.000	*p* = 0.023	*p* = 0.254
Observations	305	304	304
*R* ^2^	0.003	0.110	0.307
Adjusted *R*^2^	−0.0005	0.095	0.290
Residual Std. Error	10.560 (df = 303)	10.053 (df = 298)	8.903 (df = 296)
*F* statistic	0.860 (df = 1; 303) (*p* = 0.355)	7.379^***^ (df = 5; 298) (*p* = 0.00001)	18.720^***^ (df = 7; 296) (*p* = 0.000)

Testing the fee-free educational intervention on longitudinal changes in CANTAB PAL total errors adjusted (2–8). *p < 0.1; **p < 0.05; ***p < 0.01. Unstandardized coefficient estimates are presented, with raw *p*-values provided below. For categorical variables; non-campus, prior completion of tertiary education and males are effect size estimates. PSW, propensity score weighting.

**Table 5:** Impact of ISLAND campus intervention on cognition via CANTAB spatial working memory (SWM).

	Dependent variable		
	**SWM strategy score at follow up**		
	(1)	(2)	(3)
Campus participation (Non-campus)	1.305**	1.047*	1.017**
	*p* = 0.029	*p* = 0.069	*p* = 0.046
Age		0.220***	−1.421
		p = 0.00000	*p* = 0.285
Gender (male)		−2.065***	9.263
		*p* = 0.002	*p* = 0.300
Prior completion of tertiary education		−0.966	−0.654
		*p* = 0.108	*p* = 0.217
SWM strategy score at baseline			0.482***
			p = 0.000
PSW			205.216
			*p* = 0.253
Constant	12.645***	0.259	−25.752
	*p* = 0.000	*p* = 0.916	*p* = 0.265

Observations	306	305	304
*R* ^2^	0.016	0.134	0.335
Adjusted *R*^2^	0.012	0.119	0.319
Residual Std. Error	5.078 (df = 304)	4.802 (df = 299)	4.229 (df = 296)
*F* Statistic	4.817^**^ (df = 1; 304) (*p* = 0.029)	9.223^***^ (df = 5; 299) (*p* = 0.00000)	21.293^***^ (df = 7; 296) (*p* = 0.000)

Testing the fee-free educational intervention on longitudinal changes in CANTAB SWM strategy score. *p < 0.1; **p < 0.05; ***p < 0.01. Unstandardized coefficient estimates are presented, with raw *p*-values provided below. For categorical variables; non-campus, prior completion of tertiary education and males are effect size estimates. PSW, propensity score weighting.

### Plasma phosphorylated tau 181

A subset of participants (*n* = 231) were analyzed for plasma concentrations of phosphorylated tau 181 (p-tau 181). There was no significant association between ISLAND Campus participation and plasma concentrations of phosphorylated tau 181 (pg/mL; Supplementary Table 3; Supplementary Figure 2). This cross-sectional observation of p-tau 181 concentrations remained after adjusting for covariates.

### Stress, efficacy and literacy

All participants completed the Perceived Stress Scale (PSS, Supplementary Table 4). When compared with control (non-Campus) participants, ISLAND Campus intervention participants displayed similar PSS total scores, as well as similar responses for the 10-item instrument. There were significant differences among several domains of the New General Self-Efficacy Scale (NGSS) between intervention (ISLAND Campus) and control (non-Campus) participants (Supplementary Table 5). ISLAND Campus participants displayed significantly higher NGSS total scores, as well as higher agreement with self-efficacy sentiments on goal-attainment, overcoming barriers and overcoming challenges. There were no differences observed between intervention and control participants in health literacy, as measured by the All Aspects of Health Literacy Scale (AAHL, Supplementary Table 6).

### Post-hoc power analysis

Our post-hoc power analyses were conducted using the R (Collins et al., 2019) from the fully adjusted models, with a significance value of 0.05, giving the study a statistical power of above 0.85 for all fully adjusted GAMs, indicating this study was adequately powered to detect a medium-sized effect (coefficient estimate) of ISLAND Campus on DRP and cognitive outcomes.

## Discussion

In this large-scale prospective cohort of healthy Australian adults, we found that engaging with a formal tertiary educational intervention had a positive impact on dementia risk reduction and working memory. ISLAND Campus offered completely free formal educational degrees to any Tasmanian over 50 years of age, then measured the impact of this intervention on a variety of dementia risk measurement tools. This was a unique investigation conducted at UTAS, the only university located in Australia’s island state of Tasmania. To the best of our knowledge, this is the only longitudinal investigation of the impact of a fee-free formal tertiary educational intervention on modifiable risk factors for dementia, cognition and plasma concentrations of p-tau 181. We used a robust method of propensity score matching to show how a fee-free tertiary educational intervention for adults can have a positive impact on dementia risk factor reduction and cognition, and offers an ideal educational intervention for dementia prevention strategies in mid- to later-life.

We found ISLAND Campus participants significantly improved their scores on the DRP. Both intervention and control participants were similar at baseline, so the observed differences reported in DRP may be indicating the educational intervention had a sustained positive impact on participants adherence to modifiable risk factors for dementia. When we investigated the specific risk factors that were amenable to change, the DRP categories of BMI risk and Cognitive Activity risk displayed significant differences over time. ISLAND Campus participants showed a significant reduction in risk under these categories of the DRP, suggesting the fee-free educational intervention not only had a positive impact on engagement with cognitively stimulating activities, but also on other modifiable risk factors for dementia, potentially due to the increased engagement in participants’ own health management and literacy. Improvements in Cognitive Activity remained after adjusting for covariates such as education, suggesting Campus had an impact on Cognitive Activity beyond the bounds of tertiary education.

Compared to controls, ISLAND Campus participants significantly improved their SWM over 2 years yet we observed no changes in PAL. Engagement with formal tertiary education is multifaceted, involving planning, problem solving and mental organization, all skills that are linked to SWM and the prefrontal cortex (Yue et al., 2024). ISLAND Campus participants also may have been challenged by the navigation of digital platforms, academic schedule management and multitasking which may have selectively recruited SWM abilities. We did not observe changes in PAL over time, likely due to this task’s reliance on hippocampal function (Van Dijk et al., 2010). Previous research shows general age-related decline of hippocampal function and PAL (Lee et al., 2013), therefore the ISLAND Campus intervention may not have been running long enough to see improvements in these trajectories. Our results were also similar to previous studies such as those in the THBP (Bindoff et al., 2021), where we also found cognitive improvements over time regardless of age, with the domains most amenable to change being language, verbal learning and episodic memory. Amid a global movement of mid- to later- life individuals continuing with education throughout the lifecourse (Isopahkala-Bouret et al., 2018), age is unlikely to be a barrier to engagement in mid- to later- life education as a public health intervention.

We observed strong uptake of the intervention which was available to all ISLAND participants, thus greatly reducing socioeconomic and geographical barriers to participation. When comparing ISLAND Campus (intervention) and non-Campus (control) participants, there were no significant differences in age, gender, IRSAD decile, remoteness area, total education in years, memory complaints, family history of dementia or APOE presence. Intervention participants were aged between 50 and 86 years, showing age was no barrier to participation in this tertiary educational intervention. The only significant baseline difference observed between intervention and control participants was prior completion of tertiary education, with ISLAND Campus participants displaying significantly higher education than their non-Campus counterparts. We believe this difference is likely due to previous tertiary education removing the stigma and barriers of subsequent tertiary educational engagement, and in future will target ISLAND participants with no prior tertiary education in Campus invitations, where the greatest gains are likely to be seen.

ISLAND Campus participants enrolled in a variety of courses offered at UTAS, such as Diplomas, Bachelors, Associate Degrees, Graduate Certificates and Masters. There was substantial diversity among courses chosen, for example with uptake of Family History (*n* = 103, 20.9%), Sustainable Living (*n* = 37, 7.5%), Dementia Care (*n* = 17, 3.4%) and Music (*n* = 7, 1.4%). Given growing evidence for digital literacy, online access and device ownership increasing in older adults (Morrison et al., 2023), Campus offered a unique and accessible intervention to an engaged group of participants. With less pressure to select tertiary courses that offer subsequent employment opportunities, we may be observing the impact of education at a stage of life where improving and maintaining quality of life is more of a priority than employment opportunity. We did not observe any significant baseline differences in p-tau181, although given the cross-sectional nature of our biological analysis method, we may expect to see changes in subsequent biennial rounds of biomarker sampling. Previous research has shown even in cognitively heterogenous cohorts, plasma p-tau 181 shows relative stability over 2 years (Karikari et al., 2021), therefore to observe an impact of a cognitively stimulating intervention in mid- to later- life, longitudinal analysis is necessary.

ISLAND Campus participants were similar to non-Campus participants in terms of stress perception and health literacy, however we did observe significantly higher scores for self-efficacy in intervention participants, indicating stronger sentiment for goal attainment, overcoming barriers and overcoming challenges. It may be the fee-free tertiary education was easier to engage with for individuals who had clear goals and motivations for achieving those goals. ISLAND is a public health campaign, with nested interventions designed to bring about positive dementia risk reduction activities in the community. One of the main aims of the 10-year ISLAND Project is to build participants’ self-efficacy to tackle dementia risk management (Bartlett et al., 2023; Bartlett et al., 2022). Through ISLAND Campus, we may be observing how educational interventions have capacity to build knowledge and an ability to appraise evidence and experience in tackling new opportunities for self-development and growth. For the remainder of the ISLAND Project, we hope to investigate the sustained impact of Campus on building self-efficacy, resilience and health literacy in the existing cohort, as well as offer an additional enrolment window for participants who may have missed out on the original opportunity. This will also provide opportunity to recruit a more diverse cross-section of Tasmania, with intervention efforts primarily targeted toward those without prior history of tertiary level education.

There are numerous strengths to this unique study. ISLAND Campus was a longitudinal initiative offered to all ISLAND participants, with minimal exclusion criteria; any Tasmanian over 50 was eligible to enrol. ISLAND Campus was designed to reduce socioeconomic and geographical barriers to participation in education, we saw no impact of geographic location or socioeconomic status on participation. Building on our previous results from the THBP (Bindoff et al., 2021), ISLAND Campus was offered via flexible in-person/online learning models, that were more readily available as a result of the COVID-19 pandemic (Bartlett et al., 2021). This study addressed gaps in equity, where traditional hierarchical models of family archetypes and career trajectories may have prevented participants from exploring tertiary education earlier in life (Machů et al., 2022). Further, by offering fee-free tertiary degrees to anyone over 50, ISLAND Campus removed the socioeconomic incentive to undertake formal tertiary education, instead advocating for education as a tool for cognitive stimulation and enjoyment. Future endeavors to replicate our findings should strive for equity in educational offerings, to ensure the benefits to brain health equity are available to diverse populations. Our analytic technique of propensity score matching provided a robust effect of the intervention on observational longitudinal data, without requiring random assignment of intervention (Olmos and Govindasamy, 2015) Further, given the breadth and scope of ISLAND survey tools and data collection (Bartlett et al., 2022), we were able to statistically account for a multitude of covariates that may have impacted our results, including the propensity weightings calculated to compare intervention and control participants.

There are several limitations to note. We observed ISLAND Campus intervention participants had significantly higher prior history of tertiary education than their control counterparts. However, this was accounted for in all subsequent analyses, and did not impact our main results. Given Campus participants were anonymous from general university students, we were unable to account for the standardization of the educational experience. However, identifying Campus participants from general university studentship may have imparted implicit bias that may have impacted our results. Further, as this was an opt-in, non-random intervention, we are unable to claim causation, yet our method of propensity score matching and weighting based on age and gender provides a robust estimate of the effect of the intervention, without requiring random allocation. We acknowledge several of our measures were self-reported in nature and may have imparted a bias to our findings, particularly with respect to the annual DRP. Further, several measures were cross-sectional, including our psychosocial surveys (PSS, NGSS, AAHL), however longitudinal data collection is underway.

Previous research has classified education as an early life modifiable risk factor for dementia (Livingston et al., 2020), limiting the modeling of educational interventions in mid- to later-life (Mukadam et al., 2020). ISLAND Campus demonstrates how education is an endeavor throughout the lifecourse, and that educational interventions can have efficacy in mid- to later-life as well as the traditional model of education in early life. Here we have shown how a longitudinal formal tertiary educational intervention open to anyone in the community over 50 years of age can have an equitable, sustained and positive impact on dementia risk reduction through modifiable risk factors and cognition. With biennial biomarker collection planned, we are interested to see whether fee-free formal tertiary education can potentially reduce the hallmark biomarkers of dementia, or mitigate the neuropathological effects of biomarkers on cognitive capacity.

## Data Availability

The original contributions presented in the study are included in the article/supplementary material, further inquiries can be directed to the corresponding author.
