# Supplementary material for: ISLAND Campus: a fee-free formal university educational intervention in mid- to later-life to reduce modifiable risk factors for dementia and improve cognition
Source: Front Aging Neurosci. 2024 Dec 5;16:1479926. doi: 10.3389/fnagi.2024.1479926 (PMC11656078; doi:10.3389/fnagi.2024.1479926)
Supplement: Supplementary file 1 [file Table_1.DOCX]

|  | **Campus Participant** | **ISLAND Participant** | **Overall** |  | **P-value** |
| --- | --- | --- | --- | --- | --- |
|  | **(N=492)** | **(N=3249)** | **(N=3741)** |  |  |
| **Age (in years)** | |  |  |  | **<0.001** |
| Mean (SD) | 61.2 (7.31) | 64.5 (7.79) | 64.1 (7.81) |  |  |
| Median [Min, Max] | 60.0 [50.0, 86.0] | 64.0 [50.0, 91.0] | 64.0 [50.0, 91.0] | |  |
| **Gender** |  |  |  |  | 0.118 |
| Female | 351 (71.3%) | 2343 (72.1%) | 2694 (72.0%) | |  |
| Male | 138 (28.0%) | 902 (27.8%) | 1040 (27.8%) | |  |
| Other | 2 (0.4%) | 2 (0.1%) | 4 (0.1%) |  |  |
| Prefer not to say | 1 (0.2%) | 2 (0.1%) | 3 (0.1%) |  |  |
| **IRSAD Decile** | |  |  |  | 0.040 |
| Mean (SD) | 5.43 (2.84) | 5.15 (2.84) | 5.19 (2.84) |  |  |
| Median [Min, Max] | 6.00 [1.00, 10.0] | 6.00 [1.00, 10.0] | 6.00 [1.00, 10.0] | |  |
| **Remoteness Area** | |  |  |  | 0.621 |
| Inner Regional Australia | 368 (74.8%) | 2400 (73.9%) | 2768 (74.0%) | |  |
| Outer Regional Australia | 118 (24.0%) | 814 (25.1%) | 932 (24.9%) |  |  |
| Remote Australia | 3 (0.6%) | 9 (0.3%) | 12 (0.3%) |  |  |
| Very Remote Australia | 2 (0.4%) | 11 (0.3%) | 13 (0.3%) |  |  |
| **Total years of school** | |  |  |  | 0.008 |
| Mean (SD) | 11.7 (1.34) | 11.5 (1.46) | 11.5 (1.45) |  |  |
| Median [Min, Max] | 12.0 [4.00, 20.0] | 12.0 [0, 20.0] | 12.0 [0, 20.0] | |  |
| **Highest level of education obtained** | | | | | **<0.001** |
| Bachelor’s Degree | 121 (24.6%) | 707 (21.8%) | 828 (22.1%) |  |  |
| Certificate or Apprenticeship (including Cert 2, 3 or 4) | 37 (7.5%) | 332 (10.2%) | 369 (9.9%) |  |  |
| Diploma / Associate Degree | 73 (14.8%) | 589 (18.1%) | 662 (17.7%) |  |  |
| High School | 26 (5.3%) | 435 (13.4%) | 461 (12.3%) |  |  |
| Higher University degree (Honours, Graduate Diploma, Masters or PhD) | 215 (43.7%) | 1029 (31.7%) | 1244 (33.3%) | |  |
| Other | 16 (3.3%) | 95 (2.9%) | 111 (3.0%) |  |  |
| **Prior completion of tertiary education** | | |  |  | **<0.001** |
| No | 117 (23.8%) | 1231 (37.9%) | 1348 (36.0%) | |  |
| Yes | 374 (76.0%) | 1948 (60.0%) | 2322 (62.1%) | |  |
| **Have you noticed a substantial change in your memory and mental function in recent years?** | | | | | 0.859 |
| No | 400 (81.3%) | 2606 (80.2%) | 3006 (80.4%) | |  |
| Yes | 88 (17.9%) | 591 (18.2%) | 679 (18.2%) |  |  |
| **Is there a history of conditions such as dementia in your direct family for example siblings, parents, grandparents, aunties and uncles?** | | | | | 0.447 |
| No | 272 (55.3%) | 1676 (51.6%) | 1948 (52.1%) | |  |
| Yes | 217 (44.1%) | 1520 (46.8%) | 1737 (46.4%) | |  |
| **Apolipoprotein E (APOE) e4 Presence** | | |  |  | 0.622 |
| No | 180 (36.6%) | 835 (25.7%) | 1015 (27.1%) | |  |
| Yes | 57 (11.6%) | 291 (9.0%) | 348 (9.3%) |  |  |

**SUPPLEMENTARY TABLE 1**

| **Baseline** | | | | **Follow-up** | | |
| --- | --- | --- | --- | --- | --- | --- |
|  | **Campus Participant** | **Not Campus Participant** | **P-value** | **Campus Participant** | **Not Campus Participant** | **P-value** |
|  | **(N=492)** | **(N=492)** |  | **(N=492)** | **(N=492)** |  |
| **Alcohol risk** | |  |  |  |  |  |
| high | 61 (12.4%) | 63 (12.8%) | 0.856 | 42 (8.5%) | 47 (9.6%) | 0.392 |
| low | 249 (50.6%) | 244 (49.6%) |  | 298 (60.6%) | 283 (57.5%) |  |
| medium | 157 (31.9%) | 167 (33.9%) |  | 133 (27.0%) | 150 (30.5%) |  |
| unknown | 25 (5.1%) | 18 (3.7%) |  | 19 (3.9%) | 12 (2.4%) |  |
| **Blood Pressure risk** | |  |  |  |  |  |
| high | 39 (7.9%) | 30 (6.1%) | 0.392 | 31 (6.3%) | 17 (3.5%) | 0.070 |
| low | 441 (89.6%) | 451 (91.7%) |  | 450 (91.5%) | 459 (93.3%) |  |
| medium | 12 (2.4%) | 9 (1.8%) |  | 11 (2.2%) | 16 (3.3%) |  |
| unknown | 0 (0%) | 2 (0.4%) |  |  |  |  |
| **BMI risk** |  |  |  |  |  |  |
| high | 123 (25.0%) | 137 (27.8%) | 0.198 | 114 (23.2%) | 146 (29.7%) | 0.025 |
| low | 186 (37.8%) | 186 (37.8%) |  | 194 (39.4%) | 197 (40.0%) |  |
| medium | 177 (36.0%) | 158 (32.1%) |  | 184 (37.4%) | 146 (29.7%) |  |
| unknown | 6 (1.2%) | 11 (2.2%) |  | 0 (0%) | 3 (0.6%) |  |
| **Cholesterol risk** | |  |  |  |  |  |
| high | 89 (18.1%) | 86 (17.5%) | 0.575 | 53 (10.8%) | 64 (13.0%) | 0.183 |
| low | 385 (78.3%) | 379 (77.0%) |  | 403 (81.9%) | 402 (81.7%) |  |
| medium | 17 (3.5%) | 23 (4.7%) |  | 36 (7.3%) | 25 (5.1%) |  |
| unknown | 1 (0.2%) | 4 (0.8%) |  | 0 (0%) | 1 (0.2%) |  |
| **Diabetes risk** | |  |  |  |  |  |
| high | 88 (17.9%) | 78 (15.9%) | 0.551 | 58 (11.8%) | 62 (12.6%) | 0.560 |
| low | 394 (80.1%) | 404 (82.1%) |  | 426 (86.6%) | 424 (86.2%) |  |
| medium | 9 (1.8%) | 7 (1.4%) |  | 7 (1.4%) | 5 (1.0%) |  |
| unknown | 1 (0.2%) | 3 (0.6%) |  | 1 (0.2%) | 1 (0.2%) |  |
| **Diet risk** |  |  |  |  |  |  |
| high | 32 (6.5%) | 32 (6.5%) | 0.408 | 17 (3.5%) | 22 (4.5%) | 0.319 |
| low | 91 (18.5%) | 97 (19.7%) |  | 160 (32.5%) | 134 (27.2%) |  |
| medium | 369 (75.0%) | 361 (73.4%) |  | 312 (63.4%) | 331 (67.3%) |  |
| unknown | 0 (0%) | 2 (0.4%) |  | 3 (0.6%) | 5 (1.0%) |  |
| **Cognitive Activity risk** | |  |  |  |  |  |
| high | 205 (41.7%) | 204 (41.5%) | 0.559 | 67 (13.6%) | 118 (24.0%) | **<0.001** |
| low | 284 (57.7%) | 283 (57.5%) |  | 417 (84.8%) | 368 (74.8%) |  |
| unknown | 3 (0.6%) | 5 (1.0%) |  | 8 (1.6%) | 6 (1.2%) |  |
| **Physical Activity risk** | |  |  |  |  |  |
| high | 50 (10.2%) | 52 (10.6%) | 0.877 | 25 (5.1%) | 30 (6.1%) | 0.811 |
| low | 435 (88.4%) | 438 (89.0%) |  | 463 (94.1%) | 460 (93.5%) |  |
| unknown | 7 (1.4%) | 2 (0.4%) |  | 4 (0.8%) | 2 (0.4%) |  |
| **Smoking risk** | |  |  |  |  |  |
| high | 16 (3.3%) | 27 (5.5%) | 0.322 | 12 (2.4%) | 22 (4.5%) | 0.225 |
| low | 469 (95.3%) | 458 (93.1%) |  | 473 (96.1%) | 461 (93.7%) |  |
| medium | 7 (1.4%) | 7 (1.4%) |  | 6 (1.2%) | 7 (1.4%) |  |
| unknown |  |  |  | 1 (0.2%) | 2 (0.4%) |  |

**SUPPLEMENTARY TABLE 2**

|  | | | |
| --- | --- | --- | --- |
|  | | | |
|  | Dependent variable: | | |
|  |  | | |
|  | Plasma phosphorylated tau 181 (pg/ml) | | |
|  | (1) | (2) | (3) |
|  | | | |
| Campus participation | 0.044 | 0.069 | 0.074 |
|  | (0.088) | (0.086) | (0.085) |
| Age |  | 0.028^***^ | 0.471^**^ |
|  |  | (0.006) | (0.239) |
| Gender |  | 0.098 | -2.870^*^ |
|  |  | (0.084) | (1.600) |
| Prior tertiary education |  | 0.041 | 0.032 |
|  |  | (0.086) | (0.086) |
| PSW |  |  | -59.697^*^ |
|  |  |  | (32.132) |
| Constant | 1.369^***^ | -0.452 | 7.155^*^ |
|  | (0.050) | (0.361) | (4.110) |
|  | | | |
| Observations | 231 | 229 | 229 |
| R^2^ | 0.001 | 0.110 | 0.124 |
| Adjusted R^2^ | -0.003 | 0.095 | 0.104 |
| Residual Std. Error | 0.629 (df = 229) | 0.599 (df = 224) | 0.595 (df = 223) |
| F Statistic | 0.250 (df = 1; 229) (p = 0.618) | 6.953^***^ (df = 4; 224) (p = 0.00003) | 6.314^***^ (df = 5; 223) (p = 0.00002) |
|  | | | |
| Note: | ^*^p<0.1; ^**^p<0.05; ^***^p<0.01 | | |

**SUPPLEMENTARY TABLE 3**

|  | **Campus Participant** | **Not Campus Participant** | **P-value** |
| --- | --- | --- | --- |
|  | **(N=492)** | **(N=492)** |  |
| **In the last month, how often have you been upset because of something that happened unexpectedly?** | | | |
| Never | 72 (14.6%) | 64 (13.0%) | 0.475 |
| Almost never | 183 (37.2%) | 176 (35.8%) |  |
| Sometimes | 178 (36.2%) | 204 (41.5%) |  |
| Fairly often | 50 (10.2%) | 41 (8.3%) |  |
| Very often | 9 (1.8%) | 7 (1.4%) |  |
| **In the last month, how often have you felt that you were unable to control the important things in your life?** | | | |
| Never | 87 (17.7%) | 85 (17.3%) | 0.830 |
| Almost never | 183 (37.2%) | 177 (36.0%) |  |
| Sometimes | 163 (33.1%) | 175 (35.6%) |  |
| Fairly often | 49 (10.0%) | 42 (8.5%) |  |
| Very often | 10 (2.0%) | 13 (2.6%) |  |
| **In the last month, how often have you felt nervous and stressed?** | | | |
| Never | 52 (10.6%) | 49 (10.0%) | 0.774 |
| Almost never | 178 (36.2%) | 163 (33.1%) |  |
| Sometimes | 188 (38.2%) | 208 (42.3%) |  |
| Fairly often | 59 (12.0%) | 57 (11.6%) |  |
| Very often | 15 (3.0%) | 15 (3.0%) |  |
| **In the last month, how often have you felt confident about your ability to handle your personal problems?** | | | |
| Never | 12 (2.4%) | 4 (0.8%) | 0.127 |
| Almost never | 18 (3.7%) | 28 (5.7%) |  |
| Sometimes | 77 (15.7%) | 74 (15.0%) |  |
| Fairly often | 189 (38.4%) | 203 (41.3%) |  |
| Very often | 196 (39.8%) | 183 (37.2%) |  |
| **In the last month, how often have you felt that things were going your way?** | | | |
| Never | 2 (0.4%) | 3 (0.6%) | 0.989 |
| Almost never | 28 (5.7%) | 30 (6.1%) |  |
| Sometimes | 134 (27.2%) | 134 (27.2%) |  |
| Fairly often | 229 (46.5%) | 229 (46.5%) |  |
| Very often | 99 (20.1%) | 96 (19.5%) |  |
| **In the last month, how often have you found that you could not cope with all the things that you had to do?** | | | |
| Never | 129 (26.2%) | 119 (24.2%) | 0.422 |
| Almost never | 193 (39.2%) | 200 (40.7%) |  |
| Sometimes | 120 (24.4%) | 115 (23.4%) |  |
| Fairly often | 42 (8.5%) | 41 (8.3%) |  |
| Very often | 8 (1.6%) | 17 (3.5%) |  |
| **In the last month, how often have you been able to control irritations in your life?** | | | |
| Never | 3 (0.6%) | 1 (0.2%) | 0.238 |
| Almost never | 21 (4.3%) | 23 (4.7%) |  |
| Sometimes | 70 (14.2%) | 93 (18.9%) |  |
| Fairly often | 255 (51.8%) | 231 (47.0%) |  |
| Very often | 143 (29.1%) | 144 (29.3%) |  |
| **In the last month, how often have you felt that you were on top of things?** | | | |
| Never | 4 (0.8%) | 4 (0.8%) | 0.380 |
| Almost never | 33 (6.7%) | 25 (5.1%) |  |
| Sometimes | 101 (20.5%) | 113 (23.0%) |  |
| Fairly often | 220 (44.7%) | 237 (48.2%) |  |
| Very often | 134 (27.2%) | 113 (23.0%) |  |
| **In the last month, how often have you been angered because of things that were outside of your control?** | | | |
| Never | 80 (16.3%) | 66 (13.4%) | 0.777 |
| Almost never | 195 (39.6%) | 199 (40.4%) |  |
| Sometimes | 177 (36.0%) | 186 (37.8%) |  |
| Fairly often | 35 (7.1%) | 37 (7.5%) |  |
| Very often | 5 (1.0%) | 4 (0.8%) |  |
| **In the last month, how often have you felt difficulties were piling up so high that you could not overcome them?** | | | |
| Never | 206 (41.9%) | 193 (39.2%) | 0.690 |
| Almost never | 178 (36.2%) | 177 (36.0%) |  |
| Sometimes | 81 (16.5%) | 98 (19.9%) |  |
| Fairly often | 22 (4.5%) | 20 (4.1%) |  |
| Very often | 5 (1.0%) | 4 (0.8%) |  |
| **PSS Total** |  |  |  |
| Mean (SD)  Median [Min, Max] | 12.1 (6.79)  12.0 [0, 36.0] | 12.4 (6.53)  12.0 [0, 36.0] | 0.421 |

**SUPPLEMENTARY TABLE 4**

|  | **Campus Participant** | **Not Campus Participant** | **P-value** |
| --- | --- | --- | --- |
|  | **(N=492)** | **(N=492)** |  |
| **I will be able to achieve most of the goals that I have set for myself** | | | |
| Strongly disagree | 8 (1.6%) | 10 (2.0%) | **0.00248** |
| Disagree | 7 (1.4%) | 8 (1.6%) |  |
| Neither agree nor disagree | 25 (5.1%) | 45 (9.1%) |  |
| Agree | 284 (57.7%) | 312 (63.4%) |  |
| Strongly agree | 168 (34.1%) | 117 (23.8%) |  |
| **When facing difficult tasks, I am certain that I will accomplish them** | | | |
| Strongly disagree | 3 (0.6%) | 2 (0.4%) | 0.646 |
| Disagree | 17 (3.5%) | 17 (3.5%) |  |
| Neither agree nor disagree | 69 (14.0%) | 82 (16.7%) |  |
| Agree | 312 (63.4%) | 314 (63.8%) |  |
| Strongly agree | 91 (18.5%) | 77 (15.7%) |  |
| **In general, I think that I can obtain outcomes that are important to me** | | | |
| Strongly disagree | 2 (0.4%) | 4 (0.8%) | 0.0667 |
| Disagree | 4 (0.8%) | 4 (0.8%) |  |
| Neither agree nor disagree | 12 (2.4%) | 21 (4.3%) |  |
| Agree | 307 (62.4%) | 333 (67.7%) |  |
| Strongly agree | 167 (33.9%) | 130 (26.4%) |  |
| **I believe I can succeed at most any endeavor to which I set my mind** | | | |
| Strongly disagree | 3 (0.6%) | 2 (0.4%) | 0.721 |
| Disagree | 8 (1.6%) | 12 (2.4%) |  |
| Neither agree nor disagree | 48 (9.8%) | 60 (12.2%) |  |
| Agree | 296 (60.2%) | 291 (59.1%) |  |
| Strongly agree | 107 (21.7%) | 97 (19.7%) |  |
| **I will be able to successfully overcome many challenges** | | | |
| Strongly disagree | 2 (0.4%) | 2 (0.4%) | **0.0198** |
| Disagree | 2 (0.4%) | 11 (2.2%) |  |
| Neither agree nor disagree | 35 (7.1%) | 55 (11.2%) |  |
| Agree | 347 (70.5%) | 322 (65.4%) |  |
| Strongly agree | 106 (21.5%) | 102 (20.7%) |  |
| **I am confident that I can perform effectively on many different tasks** | | | |
| Strongly disagree | 3 (0.6%) | 4 (0.8%) | 0.107 |
| Disagree | 2 (0.4%) | 6 (1.2%) |  |
| Neither agree nor disagree | 32 (6.5%) | 51 (10.4%) |  |
| Agree | 333 (67.7%) | 324 (65.9%) |  |
| Strongly agree | 122 (24.8%) | 107 (21.7%) |  |
| **Compared to other people, I can do most tasks very well** | | | |
| Strongly disagree | 2 (0.4%) | 2 (0.4%) | 0.326 |
| Disagree | 8 (1.6%) | 16 (3.3%) |  |
| Neither agree nor disagree | 187 (38.0%) | 193 (39.2%) |  |
| Agree | 246 (50.0%) | 223 (45.3%) |  |
| Strongly agree | 49 (10.0%) | 58 (11.8%) |  |
| **Even when things are tough, I can perform quite well** | | | |
| Strongly disagree | 2 (0.4%) | 2 (0.4%) | 0.578 |
| Disagree | 10 (2.0%) | 13 (2.6%) |  |
| Neither agree nor disagree | 71 (14.4%) | 88 (17.9%) |  |
| Agree | 343 (69.7%) | 322 (65.4%) |  |
| Strongly agree | 66 (13.4%) | 67 (13.6%) |  |
| **Total NGS Score (8-40)** | |  |  |
| Mean (SD)  Median [Min, Max] | 32.2 (3.98)  32.0 [8.00, 40.0] | 31.6 (4.38)  31.0 [8.00, 40.0] | **0.0209** |

**SUPPLEMENTARY TABLE 5**

|  | **Campus Participant** | **Not Campus Participant** | **P-value** |
| --- | --- | --- | --- |
|  | **(N=492)** | **(N=492)** |  |
| **How often do you need someone to help you when you are given information to read by your doctor, nurse or pharmacist?** | | | |
| Rarely | 477 (97.0%) | 479 (97.4%) | 0.27 |
| Sometimes | 15 (3.0%) | 11 (2.2%) |  |
| Often | 0 (0%) | 2 (0.4%) |  |
| **When you need help, can you easily get hold of someone to assist you?** | | | |
| Rarely | 4 (0.8%) | 5 (1.0%) | 0.681 |
| Sometimes | 37 (7.5%) | 47 (9.6%) |  |
| Often | 300 (61.0%) | 289 (58.7%) |  |
| **Do you need help to fill in official documents?** | | | |
| Rarely | 462 (93.9%) | 471 (95.7%) | 0.263 |
| Sometimes | 24 (4.9%) | 19 (3.9%) |  |
| Often | 6 (1.2%) | 2 (0.4%) |  |
| **When you talk to a doctor or nurse, do you give them all the information they need to help you?** | | | |
| Rarely | 7 (1.4%) | 4 (0.8%) | 0.0825 |
| Sometimes | 37 (7.5%) | 56 (11.4%) |  |
| Often | 448 (91.1%) | 432 (87.8%) |  |
| **When you talk to a doctor or nurse, do you ask the questions you need to ask?** | | | |
| Rarely | 6 (1.2%) | 4 (0.8%) | 0.797 |
| Sometimes | 57 (11.6%) | 55 (11.2%) |  |
| Often | 429 (87.2%) | 433 (88.0%) |  |
| **When you talk to a doctor or nurse, do you make sure they explain anything you do not understand?** | | | |
| Rarely | 27 (5.5%) | 26 (5.3%) | 0.751 |
| Sometimes | 148 (30.1%) | 159 (32.3%) |  |
| Often | 317 (64.4%) | 307 (62.4%) |  |
| **Are you someone who likes to find out lots of information about your health?** | | | |
| Rarely | 9 (1.8%) | 8 (1.6%) | 0.706 |
| Sometimes | 68 (13.8%) | 77 (15.7%) |  |
| Often | 415 (84.3%) | 407 (82.7%) |  |
| **How often do you think carefully about whether health information makes sense in your particular situation?** | | | |
| Rarely | 0 (0%) | 1 (0.2%) | 0.485 |
| Sometimes | 28 (5.7%) | 33 (6.7%) |  |
| Often | 464 (94.3%) | 458 (93.1%) |  |
| **How often do you try to work out whether information about your health can be trusted?** | | | |
| Rarely | 59 (12.0%) | 84 (17.1%) | 0.0768 |
| Sometimes | 190 (38.6%) | 181 (36.8%) |  |
| Often | 243 (49.4%) | 227 (46.1%) |  |
| **Are you the sort of person who might question your doctor or nurse's advice based on your own research?** | | | |
| Not really | 76 (15.4%) | 103 (20.9%) | 0.0547 |
| Maybe / sometimes | 240 (48.8%) | 212 (43.1%) |  |
| Yes, definitely | 176 (35.8%) | 177 (36.0%) |  |
| **Do you think there are plenty of ways to have a say in what the government does about health?** | | | |
| Not really | 198 (40.2%) | 203 (41.3%) | 0.439 |
| Maybe / sometimes | 241 (49.0%) | 225 (45.7%) |  |
| Yes, definitely | 53 (10.8%) | 64 (13.0%) |  |
| **In the last 12 months have you taken action to do something about a health issue that affects your family or community?** | | | |
| No | 200 (40.7%) | 220 (44.7%) | 0.221 |
| Yes | 292 (59.3%) | 272 (55.3%) |  |
| **What do you think matters most for everyone's health?** | | | |
| Good housing, education, decent jobs and good local facilities | 379 (77.0%) | 360 (73.2%) | 0.185 |
| Information and encouragement to lead healthy lifestyles | 113 (23.0%) | 132 (26.8%) |  |
| **Literacy Sub Score: Functional** | | |  |
| Mean (SD) | 8.14 (2.47) | 8.33 (3.06) | 0.297 |
| Median [Min, Max] | 9.00 [4.00, 36.0] | 9.00 [5.00, 32.0] | |
| **Literacy Sub Score: Communicative** | | |  |
| Mean (SD) | 8.63 (2.53) | 8.82 (3.32) | 0.321 |
| Median [Min, Max] | 9.00 [3.00, 36.0] | 9.00 [4.00, 36.0] | |
| **Literacy Sub Score: Critical** | | |  |
| Mean (SD) | 10.7 (3.18) | 10.8 (3.83) | 0.849 |
| Median [Min, Max] | 11.0 [6.00, 40.0] | 10.0 [5.00, 45.0] | |
| **Literacy Total Score** | |  |  |
| Mean (SD)  Median [Min, Max] | 26.6 (2.39)  27.0 [17.0, 30.0] | 26.4 (2.58)  27.0 [15.0, 30.0] | 0.219 |

**SUPPLEMENTARY TABLE 6**
